# *Fritillaria pallidiflora* Schrenk ex Fisch. & C.A.Mey. (Yi Beimu): Ethnopharmacology, Phytochemistry, Pharmacological Insights, and Future Prospects

**DOI:** 10.3390/plants14243771

**Published:** 2025-12-11

**Authors:** Kailibinuer Aierken, Jinyao Li, Abdul Waheed

**Affiliations:** 1 Xinjiang Key Laboratory of Biological Resources and Genetic Engineering, College of Life Science and Technology, Xinjiang University, Urumqi 830017, China; kailibinueraierken@163.com; 2 State Key Laboratory of Desert and Oasis Ecology, Xinjiang Institute of Ecology and Geography, Chinese Academy of Sciences, Urumqi 830011, China; drwaheed@ms.xjb.ac.cn

**Keywords:** *Fritillaria pallidiflora*, Yi Beimu, steroidal alkaloids, pharmacological activities, phytochemistry, conservation

## Abstract

*Fritillaria pallidiflora* Schrenk ex Fisch. & C.A.Mey. (Yi Beimu) is a culturally significant Beimu drug in Northwest China, officially listed in the Chinese Pharmacopoeia and traditionally used to clear heat, moisten the lung, resolve phlegm, and relieve cough and wheeze. This narrative, critical review synthesizes current evidence on ethnopharmacology, phytochemistry, pharmacology, pharmacokinetics/toxicology, and conservation of *F. pallidiflora* to support sustainable, evidence-based development. Literature was retrieved from major English and Chinese databases and screened for studies that unambiguously involved Yi Beimu or its key constituents. Ethnomedicinal records consistently support antitussive, expectorant, and anti-asthmatic use in Xinjiang and the Ili River Valley. Chemically, *F. pallidiflora* is rich in cevanine-type steroidal alkaloids (e.g., imperialine, peimine, yibeinones), steroidal saponins (pallidiflosides), polysaccharides, and minor phenolics. Preclinical data show that alkaloids relax airway smooth muscle, suppress inflammatory mediators, and contribute to antitussive and anti-asthmatic effects, while polysaccharides and total alkaloid extracts exhibit antioxidant and cytoprotective activity in cell and animal models of airway injury. Additional studies report cytotoxic saponins and seed-derived antimicrobial peptides. Pharmacokinetic work highlights low to moderate and variable oral bioavailability, shaped by P-glycoprotein efflux and CYP-mediated metabolism, and reveals potential hERG channel inhibition for peimine as a cardiac safety concern. Overharvesting and habitat loss have reduced wild resources, underscoring the need for conservation, cultivation, and marker-guided quality control. Overall, Yi Beimu shows credible ethnopharmacological rationale and promising multi-target pharmacology for respiratory disorders, but translation will require bioactivity-guided isolation coupled with PK–PD-guided in vivo studies, rigorous safety evaluation, and conservation-aware cultivation to move from traditional remedy toward validated therapeutic resource.

## 1. Introduction

**Figure 1:**
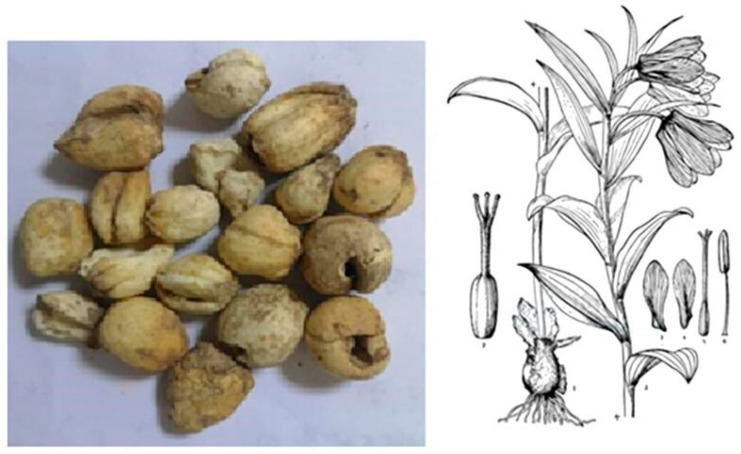
Bulbs (medicinal part) and morphological illustration of *F. pallidiflora*. The bulbs are the traditional crude drug source, while the botanical diagram shows diagnostic features of the species including leaves, flowers, and bulb morphology.

Medicinal plants remain foundational to traditional and modern healthcare world-wide [1]. Within this landscape, the genus *Fritillaria* (Liliaceae) holds particular prominence across Asian and Eurasian pharmacopeias, where dried bulbs known collectively as Beimu have been prescribed for cough suppression, phlegm clearance, and asthma for more than two millennia. Classical records in Chinese materia medica describe Beimu as a key herb for ‘clearing heat, moistening the lung, resolving phlegm, and relieving cough and wheeze’, and contemporary summaries continue to affirm its role as a cornerstone antitussive and expectorant that bridges traditional use and modern pharmacology [2]. *F. pallidiflora*, locally known as Yi Beimu, is distributed in Xinjiang and adjacent regions, with bulbs traditionally used as medicinal material (Figure 1).

While several *Fritillaria* species have been systematically studied, *Fritillaria pallidiflora* Schrenk ex Fisch. & C.A.Mey. (Yi Beimu) remains comparatively underreviewed despite growing clinical and commercial interest [3]. The bulbs of *F. pallidiflora* Schrenk and *F. walujewii* are officially recognized botanical sources of Yi Beimu in the 2020 edition of the Chinese Pharmacopoeia, anchoring their legitimacy in clinical practice and trade [4,5]. In China, *F. pallidiflora* is native to high-altitude, cool, and relatively humid niches of Xinjiang and has been cultivated more widely since the mid-twentieth century to meet demand. Recent agronomic and developmental studies further note that *F. pallidiflora* is now extensively cultivated across China and that bulb development follows recognizable thresholds that are relevant to quality control in medicinal supply chains [6].

Phytochemically, *F. pallidiflora* conforms to the genus hallmark of abundant steroidal alkaloids, along with steroidal saponins, polysaccharides, and other secondary metabolites [7]. Imperialine, peimine, and related cevanine-type isosteroidal alkaloids have been repeatedly isolated and are frequently credited with antitussive, bronchodilatory, and anti-inflammatory effects, aligning with its respiratory indications [8]. Successive investigations over the last decade have expanded the alkaloid and saponin inventory of *F. pallidiflora*, including yibeinones and pallidiflosides, while reinforcing the centrality of steroidal alkaloids to pharmacological activity [9,10]. In parallel, polysaccharide fractions from *Fritillaria* species demonstrate antioxidant and immunomodulatory activities that may complement alkaloid-driven effects and suggest multi-component synergy consistent with traditional decoction practice [11].

Despite this momentum, scholarly synthesis has not kept pace for *F. pallidiflora*. Influential reviews have focused on *F. cirrhosa* D.Don and *F. thunbergii* with detailed treatment of chemistry, biosynthesis, and therapeutic mechanisms, whereas *F. pallidiflora* is often mentioned only peripherally [12,13]. Meanwhile, primary studies specific to *F. pallidiflora* remain dispersed across phytochemical reports, isolated pharmacological assays, and emerging agronomic or omics-driven analyses, limiting cross-study comparison and translational insight [10,14,15]. This fragmentation obscures important questions that are central to modern development of Yi Beimu: which molecular classes or lead compounds best account for respiratory efficacy, what mechanisms are most plausible in vivo, how bioavailable and safe are these constituents under realistic dosing, and how can rising demand be balanced with conservation and sustainable cultivation. At the ecosystem interface, recent field and soil microbiome work in cultivation settings highlights that intensive production can reshape belowground communities, signaling the need to integrate ecological sustainability into quality and yield strategies [9].

At the same time, interest in Beimu at large continues to broaden beyond classic cough and asthma indications, with modern studies probing roles in chronic obstructive pulmonary disease, non-small cell lung cancer, and inflammation, and surveying the genus through network pharmacology and advanced analytical platforms [16]. Placing *F. pallidiflora* within this expanding context requires a focused, critical synthesis that connects its ethnomedicinal record to contemporary chemical and biological evidence and that identifies where targeted research can most productively close gaps [17].

This review integrates ethnopharmacology, phytochemistry, pharmacological activities, and future perspectives of *F. pallidiflora* to guide both scientific exploration and clinical application. Specifically, we collate and evaluate traditional usage patterns and regulatory status, synthesize the current chemical space with emphasis on steroidal alkaloids and saponins, appraise preclinical pharmacology with attention to respiratory mechanisms and adjunct activities, and outline priorities in pharmacokinetics, safety, conservation, and cultivation that can support sustainable and evidence-based development of Yi Beimu.

## 2. Materials and Methods

This review followed a narrative, critical approach with an explicit literature search strategy. We searched PubMed, Web of Science, CNKI, and Google Scholar up to December 2024 using combinations of the terms “*Fritillaria pallidiflora*”, “Yi Beimu”, “steroidal alkaloid”, “saponin”, “polysaccharide”, “antitussive”, “anti-asthmatic”, “pharmacokinetics”, “toxicity”, and “conservation”. Additional relevant articles were identified through citation tracking of key reviews and primary studies. We included original works that (i) unambiguously identified *F. pallidiflora Schrenk* ex Fisch. & C.A.Mey. or its bulbs as the study material, or (ii) reported genus-level data clearly applicable to Beimu alkaloids or polysaccharides. For each pharmacological study, we extracted information on botanical authentication (including voucher specimens where reported), plant part and origin, extraction procedures (e.g., aqueous decoction, hydroethanolic extraction, enrichment of total alkaloids or polysaccharides), and experimental models (cell lines, isolated tissues, or animal species and dosing routes). These details are summarized narratively in Section 4 to allow readers to gauge the robustness and translational relevance of the available evidence.

## 3. Taxonomy, Distribution, and Ecology of *F. pallidiflora*

*F. pallidiflora* Schrenk ex Fisch. & C.A.Mey. (Liliaceae) is a perennial herbaceous species of the genus Fritillaria, which comprises more than 150 species distributed mainly across temperate Eurasia. In the Chinese Pharmacopoeia, the bulbs of *F. pallidiflora* and *F. walujewii* are recognized as the botanical sources of Yi Beimu, distinguishing them from other Beimu categories such as Chuan Beimu (*F. cirrhosa* D.Don) and Zhe Beimu (*F. thunbergii* Miq.). Morphologically, *F. pallidiflora* is characterized by a small ovoid bulb composed of fleshy scales, a single erect stem bearing several linear-lanceolate leaves, and one to several nodding, pale yellow to yellow-green campanulate flowers.

The natural distribution of *F. pallidiflora* is centered in northwestern China and adjacent Central Asian regions [18]. In China it occurs mainly in Xinjiang, especially in the Ili, Altay and Tacheng areas, typically in forest edges, shrubs, meadows, and mountain steppe habitats at altitudes of approximately 1300–2500 m. Populations prefer cool, relatively humid microclimates with well-drained, humus-rich soils, conditions that are increasingly restricted by land-use change and overharvesting. Outside China, *F. pallidiflora* is reported from Kazakhstan and neighboring regions, where it also forms part of local medicinal floras.

Ecologically, *F. pallidiflora* shows a typical geophytic life cycle, with bulbs overwintering underground and aerial parts emerging in early spring. Its flowers provide early season nectar and pollen resources for pollinators in montane ecosystems. Recent field surveys indicate that wild populations have declined markedly in low- and mid-altitude habitats due to overcollection of bulbs and habitat disturbance, contributing to its listing as a protected species and reinforcing the need for ex situ cultivation and habitat conservation, issues that are discussed in more detail in Section 8.

## 4. Ethnopharmacology and Traditional Uses

**Figure 2:**
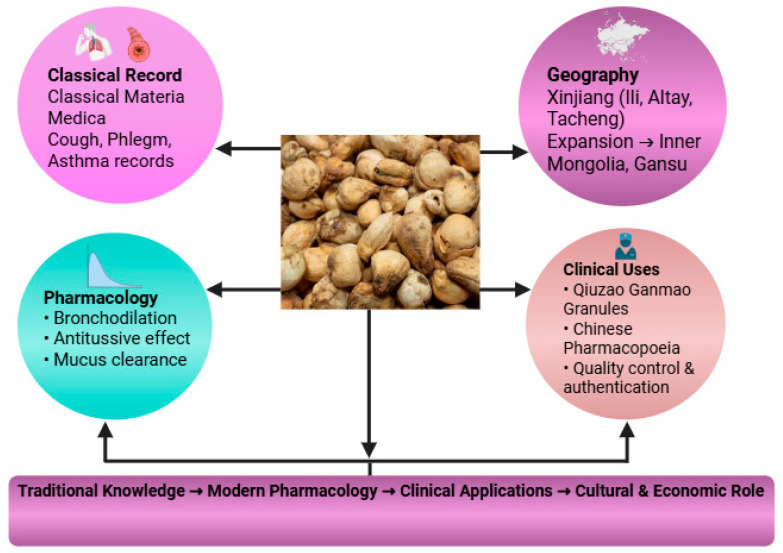
Ethnopharmacological framework of *F. pallidiflora*, highlighting classical materia medica records, regional distribution and cultivation in Xinjiang, cultural and economic significance, and recognized clinical functions in cough suppression, phlegm resolution, and asthma treatment.

Ethnopharmacological records consistently highlight the use of *F. pallidiflora* as an antitussive and expectorant, aligning with its widespread application in respiratory ailments [13]. Unlike other species, it is particularly valued in Xinjiang and the Ili River Valley, where its cultivation has become both a cultural and economic practice [19,20]. The ethnopharmacological basis of Yi Beimu encompasses its classical materia medica records, regional cultivation in Xinjiang, and recognized clinical functions in cough suppression, phlegm resolution, and asthma treatment (Figure 2). In TCM terms, these indications are summarized as ‘clearing lung heat and moistening dryness, transforming phlegm, and relieving cough and wheezing’, which provides the primary conceptual framework for clinical use of Yi Beimu.

Earliest materia medica context and regional practice. Beimu, the collective name for medicinal *Fritillaria* bulbs in Chinese materia medica, has been prescribed for cough suppression, phlegm resolution, and asthma since classical times [21]. Modern syntheses of the genus trace these respiratory indications from the historical canon into contemporary practice across East Asia and Central Asia, establishing Beimu as a cornerstone herb for airway diseases [22]. Within this lineage, *F. pallidiflora* Schrenk, together with *F. walujewii*, is recognized as the botanical origin of Yi Beimu in the 2020 Chinese Pharmacopoeia [23]. It Is native and cultivated distribution centers on high altitude and cool, humid valleys in Xinjiang, with documented expansion into Inner Mongolia and Gansu for medicinal supply, reflecting a long regional tradition of use and cultivation [24].

Consistent with Beimu’s canonical role, Yi Beimu is administered to moisten the lung, relieve cough and wheeze, resolve phlegm, and soften hard masses [25]. These indications are repeatedly noted in pharmacognostic descriptions of *F. pallidiflora* and align with modern evaluations of Fritillaria Bulbus that document cough relief, antiasthma activity, and mucus clearance as primary functional attributes [13]. Recent studies continue to bridge traditional claims with experimental observations. For example, genus level reviews and pharmacological assessments emphasize bronchodilatory and antitussive effects for Beimu drugs in preclinical models, which are congruent with historical use patterns [13,25].

In mainstream clinical practice, *F. cirrhosa* (Chuan Beimu) and *F. thunbergii* (Zhe Beimu) have received the greatest national attention and are the focus of recent comprehensive reviews, reflecting their high prescription frequency across China [26]. Yi Beimu differs in that its use is more concentrated in the northwest, where local cultivation and trade are prominent, and where it retains notable cultural value in Xinjiang clinical traditions. Historically, Chinese herbological classifications also distinguish Yi Beimu as a specific Beimu category alongside Chuan Beimu and Zhe Beimu, reinforcing its recognized identity within the materia medica system [27].

Yi Beimu appears both as a standalone crude drug and as a component of compound prescriptions. Classical decoction use targets dry cough with scanty phlegm or irritative cough with throat discomfort, often in combination with moistening and heat clearing herbs, consistent with its traditional functional profile [28]. In modern proprietary practice, Yi Beimu from *F. pallidiflora* and *F. walujewii* is documented as a key ingredient in Qiuzao Ganmao Granules, a cough and common cold formulation, which illustrates the continuity from textual tradition to regulated products. Additional contemporary work on Fritillaria Bulbus quality control, authentication, and species discrimination supports the standardized use of Beimu drugs in proprietary formulas and clinical settings [12,29].

The modern ethnobotanical landscape of Yi Beimu mirrors its geographic ecology. Sources describe longstanding cultivation in Ili, Altay, and Tacheng within Xinjiang, with expansion to Inner Mongolia and adjacent provinces to meet rising demand [30]. This pattern underscores the regional anchoring of Yi Beimu use, as well as the need to integrate cultural practice with sustainable cultivation and conservation policy [31].

Together, these lines of evidence situate Yi Beimu as a historically grounded, regionally significant Beimu drug whose traditional indications for cough, phlegm, and asthma are consistent with modern pharmacological observations, while also highlighting differences in clinical prominence and cultural valuation relative to other Fritillaria species. Its role in both traditional prescriptions and modern proprietary formulations reflects a strong cultural and medical continuity (Figure 2).

## 5. Phytochemistry of *F. pallidiflora*

**Figure 3:**
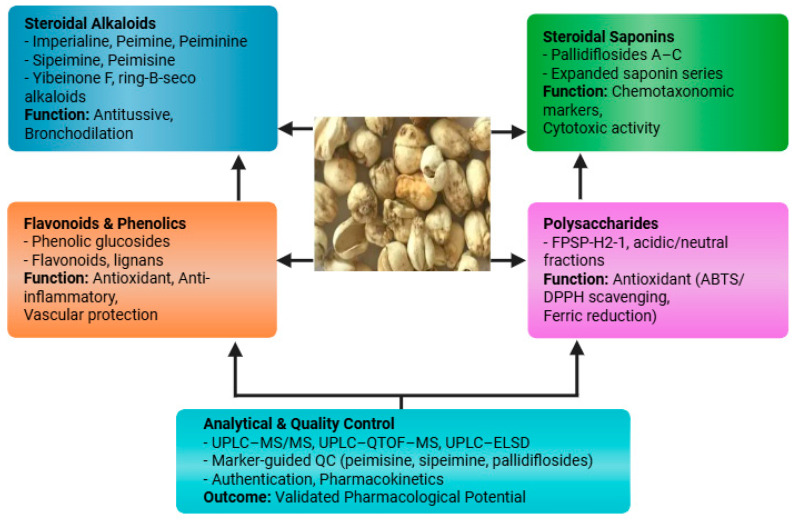
Major phytochemical classes identified in *F. pallidiflora*, including steroidal alkaloids, steroidal saponins, flavonoids/phenolics, and polysaccharides, together with representative compounds and their pharmacological associations.

The bulbs of *F. pallidiflora* contain diverse phytochemical classes, predominantly steroidal alkaloids, steroidal saponins, flavonoids/phenolics, and polysaccharides [32]. The phytochemistry of *F. pallidiflora* is characterized by four principal classes, including steroidal alkaloids, steroidal saponins, polysaccharides, and flavonoids/phenolics, each contributing to its pharmacological functions (Figure 3). These constituents underpin the plant’s traditional antitussive and expectorant use. Among the alkaloids, imperialine (verticine), peimine, and peiminine recur as key bioactives across reports, while pallidiflosides A–C and additional steroidal saponins mark a distinctive chemotaxonomic profile for Yi Beimu. Recent analytical advances (UPLC–MS/MS; UPLC–Q-TOF) have sharpened compound identification and enabled marker-guided quality control and pharmacokinetic work [33]. Our literature search identified a limited but steadily growing number of primary phytochemical investigations focused specifically on *F. pallidiflora* bulbs, which collectively define the current chemical space summarized here.

### 5.1. Steroidal Alkaloids (Hallmark Constituents)

**Figure 4:**
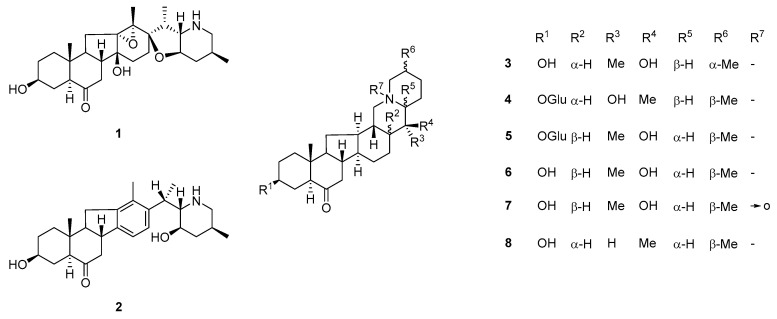
Representative steroidal alkaloid structures from *F. pallidiflora*. Structures include hallmark isosteroidal alkaloids such as imperialine (1), peimine (2), and their congeners (3–8), with R-group substitutions indicating structural diversity. These compounds represent the dominant chemical class in Yi Beimu, underpinning its traditional antitussive and bronchodilatory functions, and serve as key markers for pharmacological and quality-control studies. The bidirectional arrow in structure (7) denotes keto–enol tautomerism, indicating the equilibrium between the carbonyl and enolic hydroxyl forms commonly observed in steroidal frameworks.

Steroidal isosteroidal alkaloids are the hallmark metabolites of Beimu drugs and represent the dominant chemical group within the *F. pallidiflora* alkaloid profile [34]. Classical phytochemical investigations consistently reported major compounds such as imperialine (verticine), peimine (verticinone), peiminine, peimisine, sipeimine, and imperialine-β-D-glucoside, establishing a core set of bioactive markers that recur across collections [35]. Representative structures of these hallmark steroidal alkaloids, including imperialine, peimine, peiminine, and sipeimine, are depicted in Figure 4. These molecules, particularly imperialine and peimine, are strongly associated with the traditional antitussive and bronchodilatory effects of Yi Beimu [36].

Recent research has expanded the structural diversity of this class. For instance, yibeinone F was previously identified as a novel 24-hydroxylated cevanine-type alkaloid, together with several known congeners, and confirmed selected structures through X-ray crystallography [10]. Similarly, the discovery of five new steroidal glycoalkaloids and a rare ring-B-seco isosteroidal alkaloid, findings that highlight both the chemical novelty and biosynthetic versatility of this species [37].

Alongside isolation studies, analytical chemistry has advanced significantly. The development of a rapid UPLC–MS/MS method enabling precise quantification of sipeimine, while also mapping its tissue distribution and metabolic fate in rats [38]. Such work not only supports pharmacokinetic and ADME research but also provides tools for quality control and species authentication. In addition, broad-spectrum profiling of Xinjiang *Fritillaria* using UPLC–QTOF-MS has confirmed the abundance and complexity of alkaloid constituents in *F. pallidiflora*.

### 5.2. Steroidal Saponins (Chemotaxonomic Markers)

**Figure 5:**
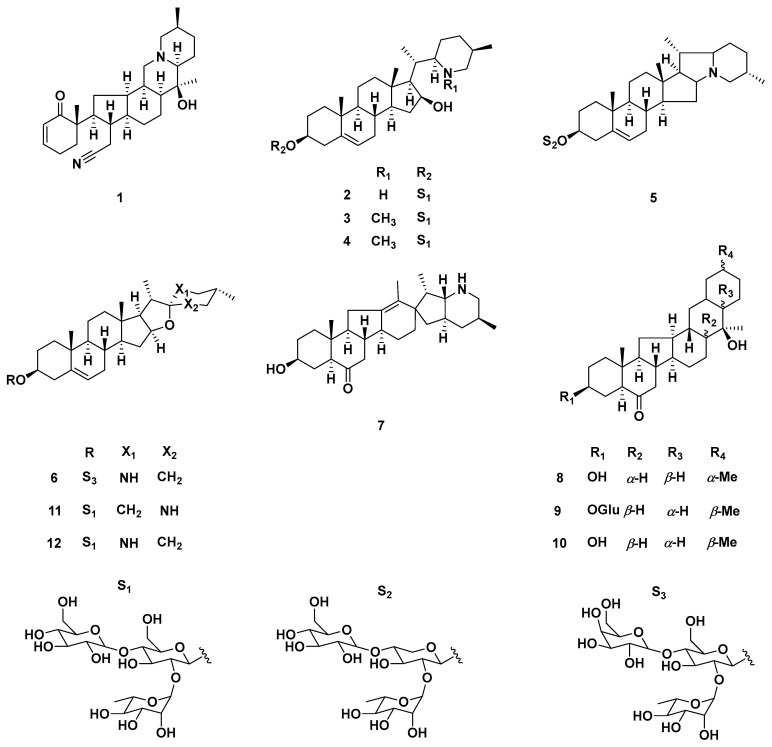
Representative steroidal saponins from *F. pallidiflora*. Structures include pallidiflosides A–C and related congeners (compounds 1–12), with variations in aglycone substitution patterns (R groups) and sugar moieties (S_1_–S_3_). These saponins serve as distinctive chemotaxonomic markers of Yi Beimu and have been reported to exhibit cytotoxic and lineage-differentiating activities. Together with steroidal alkaloids, they contribute to the phytochemical profile of *F. pallidiflora* and are important for both authentication and pharmacological evaluation.

Beyond alkaloids, *F. pallidiflora* yields a characteristic suite of steroidal saponins, led by pallidiflosides A–C first reported in 2011 and subsequently expanded (five new + seven known saponins) in a 2012 Fitoterapia study. Some of these saponins exhibited in vitro cytotoxicity (e.g., against C6 glioma and HeLa), suggesting pharmacological relevance and potential as lineage markers within the species [39,40]. The pallidifloside series, in particular, is frequently cited in authentication and comparative chemistry work to differentiate Yi Beimu from other Beimu sources [41]. Pallidiflosides and related steroidal saponins serve as key chemotaxonomic markers of *F. pallidiflora*, and their representative structures are summarized in Figure 5.

### 5.3. Flavonoids and Other Phenolics

Although present in smaller quantities compared with steroidal alkaloids and saponins, flavonoids and phenolic glucosides have been identified in *F. pallidiflora* and may contribute complementary pharmacological effects [42]. Early investigations reported two unique phenolic glucosides, representing the first such compounds described in the Liliaceae family, which drew attention to the chemical diversity of Yi Beimu beyond its hallmark alkaloid constituents [43]. Subsequent phytochemical surveys of related *Fritillaria* species have consistently documented flavonoids, phenols, and lignans in bulbs, aerial parts, or seeds, suggesting that this class of metabolites may be more widespread across the genus than initially recognized [44].

The functional significance of these minor compounds is increasingly acknowledged. Flavonoids are well known for their antioxidant, anti-inflammatory, and vascular-protective properties, raising the possibility that even trace amounts in *F. pallidiflora* could exert adjunctive effects when consumed in decoction form [45]. In particular, phenolic glucosides may act synergistically with alkaloids by stabilizing reactive oxygen species or modulating inflammatory signaling cascades, thereby reinforcing the herb’s traditional role in relieving cough and airway irritation [46].

However, the characterization of flavonoids and phenolics in Yi Beimu remains limited to a few isolated reports. Modern metabolomics approaches, such as high-resolution LC–HRMS/MS coupled with molecular networking, offer powerful tools to re-examine these fractions systematically [3]. Renewed efforts to profile flavonoids and phenolics could clarify their contribution to decoction chemistry, identify potential synergistic interactions with alkaloids and saponins, and provide additional chemotaxonomic markers for authentication and quality control [39].

### 5.4. Polysaccharides

Bulb polysaccharides of *F. pallidiflora* whose monosaccharide composition and molecular weight depend on extraction and purification show notable antioxidant capacity (ABTS/DPPH scavenging; ferric-reducing power) [47]. A widely cited sequential-extraction study resolved acidic and neutral fractions, identifying FPSP-H2-1 as a strong radical scavenger; follow-up work across *Fritillaria* spp. continues to link extraction conditions (e.g., cellulase or ultrasound assistance) to structure–activity readouts [48]. These macromolecules likely complement alkaloid-driven respiratory effects in traditional decoctions by buffering oxidative stress. Representative compounds identified from *F. pallidiflora*, their chemical classes, and reported activities are summarized in Table 1, providing an overview of the major phytochemical constituents documented to date. In addition to in vitro radical-scavenging assays, sequential polysaccharide fractions from *F. pallidiflora* have been shown to ameliorate oxidative stress markers and histopathological damage in animal models of chemically induced oxidative injury, supporting their antioxidant activity in vivo.

### 5.5. Analytical and Quality-Control Notes

For quality standardization and species authentication, multiple marker strategies have emerged. UPLC–ELSD methods simultaneously quantify peimisine and sipeimine for Yi Beimu QC; UPLC–QTOF-MS expands coverage to broader alkaloid panels in Xinjiang Fritillaria; and UPLC–MS/MS now supports bioanalytical monitoring (e.g., sipeimine PK) [58]. Representative HPLC–ELSD chromatograms of *Bulbus F. pallidiflora* reported in quality-control studies show well-resolved peaks corresponding to the major isosteroidal alkaloids (imperialine, peimine, peiminine, peimisine and sipeimine), along with smaller peaks for minor alkaloids and steroidal saponins. The overall peak pattern forms a characteristic fingerprint that helps distinguish Yi Beimu from other Beimu sources and monitor batch-to-batch consistency of decoctions, granules and alkaloid-enriched extracts. These chromatographic profiles underpin the marker-based standards adopted by the Chinese Pharmacopoeia and recent pharmacopoeial-style monographs for *Bulbus Fritillariae pallidiflorae*. Together, these platforms enable lot-level chemotyping, targeted marker release tests, and cross-species discrimination crucial where substitution with *F. cirrhosa* or other Beimu is a market risk.

Overall, the chemical space of *F. pallidiflora* is anchored by steroidal alkaloids and steroidal saponins, with polysaccharides and minor phenolics as complementary fractions. Steroidal alkaloids such as imperialine and peimine remain the hallmark constituents, while pallidiflosides and polysaccharides provide additional chemotaxonomic and functional value (Figure 3). As outlined in Table 1, these constituents include hallmark alkaloids such as imperialine and peimine, unique saponins like pallidiflosides, and polysaccharides with strong antioxidant capacity. While discovery continues evidenced by recent reports of novel yibeinones, ring-B-seco glycoalkaloids, and expanded saponin series most studies remain at the stage of isolation and structural elucidation, with limited linkage to mechanism-specific pharmacology, exposure–response relationships, or in vivo efficacy. The development of standardized marker panels (e.g., sipeimine/peimisine plus pallidiflosides) and modern bioanalytical methods now offers a bridge between chemistry and pharmacology; applying these in bioactivity-guided and pharmacokinetics-aware workflows will be essential to translate *F. pallidiflora*’s phytochemistry into validated therapeutic potential.

## 6. Pharmacological Activities

Below, activities are organized by bioactivity, explicitly linking traditional indications (cough, phlegm, asthma), the chemical classes most implicated (steroidal alkaloids, saponins, polysaccharides, peptides), and modern evidence (in vitro/in vivo where available). Where data are genus-level rather than *F. pallidiflora*–specific, this is stated clearly. In the cited preclinical work, bulbs of authenticated *F. pallidiflora* (often collected from Ili or cultivated in Xinjiang and accompanied by voucher specimens) were typically extracted with water or 70–80% ethanol, followed by fractionation to obtain total alkaloids or polysaccharides. Pharmacological activities were then evaluated in rodent models (e.g., mouse or rat) and in established cell lines such as RAW 264.7 macrophages or human bronchial epithelial cells.

### 6.1. Antitussive and Anti-Asthmatic Activity

Rationale and Link to tradition. Yi Beimu is prescribed to “moisten lung,” suppress cough, and resolve phlegm; pharmacology centered on steroidal alkaloids supports these respiratory claims [59]. In rat trachea and bronchus preparations, imperialine (verticine) and related cevanine-type alkaloids isolated from *F. pallidiflora* produce concentration-dependent relaxation of pre-contracted airway smooth muscle, aligning directly with its antitussive and bronchodilatory indications [60].

Key molecules and models. Beyond imperialine, several newly characterized cevanine-type isosteroidal alkaloids (e.g., yibeinone analogues) from *F. pallidiflora* have shown pronounced relaxant effects on isolated rat tracheal rings, complementing genus-level data that identify the major Beimu alkaloids (imperialine, peimine, verticinone and congeners) as among the most potent tracheobronchial relaxants. Mechanistically, these effects are typically assessed against carbachol- or histamine-induced contraction, suggesting modulation of cholinergic pathways and Ca^2+^ influx, which is consistent with anti-asthmatic pharmacology [61,62].

Evidence strength. The airway-relaxant signal is robust in vitro and mapped to alkaloid class chemistry but controlled in vivo cough/asthma models specific to *F. pallidiflora* remain sparse, and no clinical trials have validated dose–response or safety in humans. As such, translation is promising yet preclinical [63].

### 6.2. Anti-Inflammatory and Antioxidant Effects

Rationale and link to tradition. Chronic airway disease involves inflammation and oxidative stress; constituents of *F. pallidiflora* from multiple classes-alkaloids and polysaccharides target these axes [22].

Alkaloids (macrophage models). Steroidal alkaloids isolated from *F. pallidiflora* significantly inhibit nitric oxide (NO) in LPS-stimulated RAW264.7 macrophages; e.g., stenanzine and hapepunine showed notable potency (IC_50_ ≈ 8.0 μM and 20.9 μM, respectively), while yibeinone derivatives emerged as strong NO inhibitors in related studies [10]. These results position *F. pallidiflora* alkaloids as direct anti-inflammatory agents consistent with symptom relief in inflamed airways [64].

Polysaccharides (antioxidant capacity). *F. pallidiflora* polysaccharides—whose monosaccharide profiles and molecular weights vary by extraction exhibit ABTS/DPPH radical-scavenging and ferric-reducing activity; extraction strategy (e.g., cellulase or ultrasound assistance) modulates yield, structure, and activity [65]. These antioxidant signals support a multi-component rationale for Beimu decoctions (alkaloids + polysaccharides).

More recently, total alkaloid extracts from Bulbus *Fritillariae pallidiflorae* were shown to attenuate cigarette smoke extract–induced oxidative damage in human bronchial epithelial cells (Beas-2B), increasing total antioxidant capacity and superoxide dismutase while reducing intracellular ROS and malondialdehyde levels, effects associated with activation of SIRT1/Nrf2/Keap1 signaling and modulation of MAPK and PI3K–Akt pathways. These findings provide additional in vivo-relevant support that Yi Beimu constituents protect airway epithelium from oxidative stress, a key pathogenic driver in chronic cough and bronchial disease.

Evidence strength. Anti-inflammatory and antioxidant mechanisms are supported in vitro, with limited in vivo systems and no human data defining exposure–response or pharmacokinetics for these fractions; bioavailability of alkaloids is a known constraint for the genus.

### 6.3. Antitumor Potential

The exploration of *F. pallidiflora* for antitumor effects is relatively recent, but initial findings indicate potential for drug discovery. Steroidal saponins isolated from its bulbs have demonstrated cytotoxic activity against C6 glioma and HeLa cervical cancer cell lines, with IC_50_ values ranging between 5 and 76 μM depending on the compound [66]. In parallel, steroidal alkaloids traditionally associated with respiratory pharmacology are being assessed for anti-proliferative potential, showing selective inhibition of tumor cell viability while retaining anti-inflammatory profiles. Network pharmacology approaches have further suggested that Beimu alkaloids could target pathways associated with cell cycle arrest and apoptosis, supporting their possible role as adjuvant anticancer agents [67]. However, most of this evidence remains at the in vitro level. Studies on animal tumor models are lacking, and critical issues such as bioavailability, tumor selectivity, and toxicity thresholds have not been systematically explored. As a result, the antitumor activity of *F. pallidiflora* should be regarded as hypothesis-generating, warranting deeper investigation before translational claims can be substantiated [68].

### 6.4. Antimicrobial and Antiviral Activities

Beyond respiratory and tumor-related pharmacology, *F. pallidiflora* exhibits a novel dimension of activity through antimicrobial peptides (AMPs) found in its seeds. Research published between 2017 and 2020 has reported the isolation and characterization of AMP fractions using SDS-PAGE and LC–MS techniques, confirming bioactivity against selected bacterial and fungal strains [69]. Although these studies have used different test organisms, they consistently demonstrate that *F. pallidiflora* seeds contain peptide-based defense molecules, which expand the therapeutic landscape of Yi Beimu beyond its traditional indications. Such findings align with a broader recognition of plant AMPs as promising templates for next-generation antimicrobial agents, particularly in the context of rising antimicrobial resistance [70]. Despite these promising signals, the current body of evidence remains at an early stage, focusing on fraction-level activities without detailed structural identification or mechanism-of-action studies. Standardized antimicrobial panels, viral screening, and sequence-resolved peptide characterization are urgently needed to clarify the biomedical potential of these seed-derived components [71].

### 6.5. Other Reported Activities (Exploratory)

Across the genus, Fritillaria extracts/constituents have been explored for antidiabetic, neuroprotective, and hepatoprotective effects, but species-specific evidence for *F. pallidiflora* is limited. Recent syntheses underline multi-system potential for Beimu (e.g., COPD inflammation, tumor adjuvancy), yet rigorous *F. pallidiflora* data outside respiratory-inflammation domains are sparse and largely extrapolated from other species [13,72]. Priorities include targeted in vivo studies, exposure–response definition for lead alkaloids/saponins, and elucidation of systems-level mechanisms (e.g., network pharmacology) to guide rational indications.

Collectively, the respiratory indications for Yi Beimu map convincingly onto steroidal alkaloids that relax airway smooth muscle and dampen macrophage-mediated inflammation, with polysaccharides contributing antioxidant and cytoprotective support. At a mechanistic level, this combination of direct bronchodilation, anti-inflammatory signaling (e.g., suppression of NO/iNOS and MAPK pathways), and mitigation of oxidative stress offers a coherent explanation for its classical use to ‘clear heat, moisten the lung, resolve phlegm, and relieve cough [38]. The discovery of structurally unusual isosteroidal alkaloids (e.g., yibeinone derivatives) and ring-B-seco glycoalkaloids, together with bioactive steroidal saponins and seed-derived antimicrobial peptides, expands the pharmacological repertoire of *F. pallidiflora* and provides innovative lead structures for respiratory and anti-infective drug development [73].

**Figure 6:**
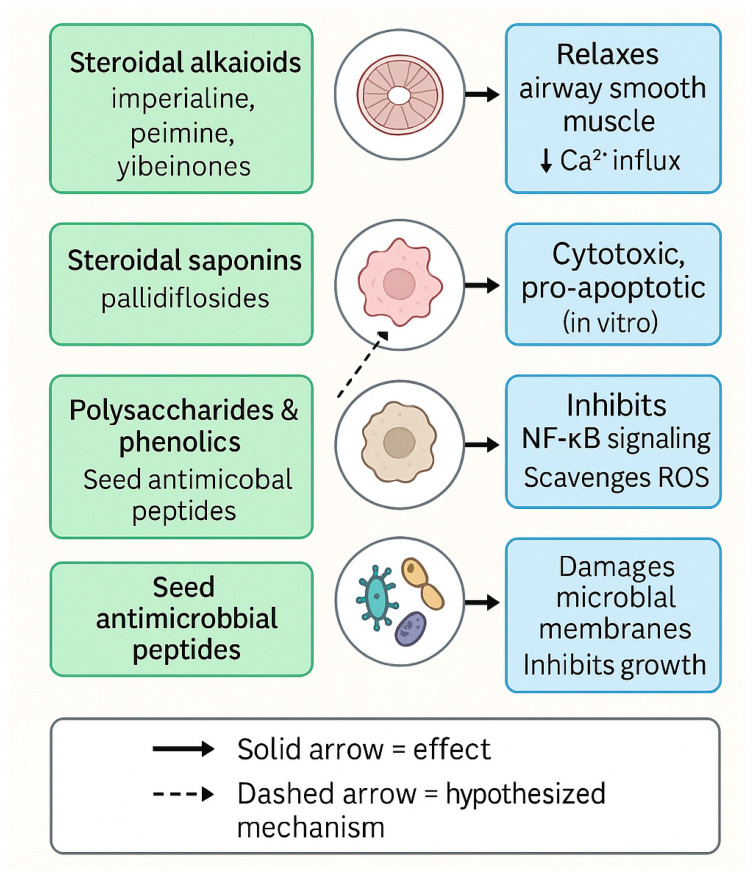
Proposed mechanisms of action underlying the major pharmacological activities of *Fritillaria pallidiflora* (Yi Beimu).

Taken together, these activities indicate that *F. pallidiflora* is not only a traditional cough and phlegm-relieving herb, but also a promising multi-component, multi-target resource with potential impact in chronic airway disease, inflammation-driven pathologies, and possibly oncology and infectious disease. The innovative aspects of Yi Beimu research include (i) the identification of new cevanine-type and ring-B-seco alkaloids with potent tracheal relaxant or anti-inflammatory activity, (ii) the recognition of polysaccharides and total alkaloid extracts as modulators of oxidative stress and epithelial injury in contemporary lung-injury models, and (iii) the emerging characterization of antimicrobial peptides from seeds. However, most of these signals are currently supported by in vitro or early preclinical work. Priorities for future research therefore include bioactivity-guided isolation linked to defined respiratory and inflammatory models, PK–PD-guided in vivo efficacy trials, and rigorous safety evaluations, which together will determine the real therapeutic impact of *F. pallidiflora* constituents. These mechanistic relationships between specific compound classes, molecular targets, and pharmacological effects are summarized schematically in Figure 6. 

## 7. Pharmacokinetics, Bioavailability, and Toxicology

**Figure 7:**
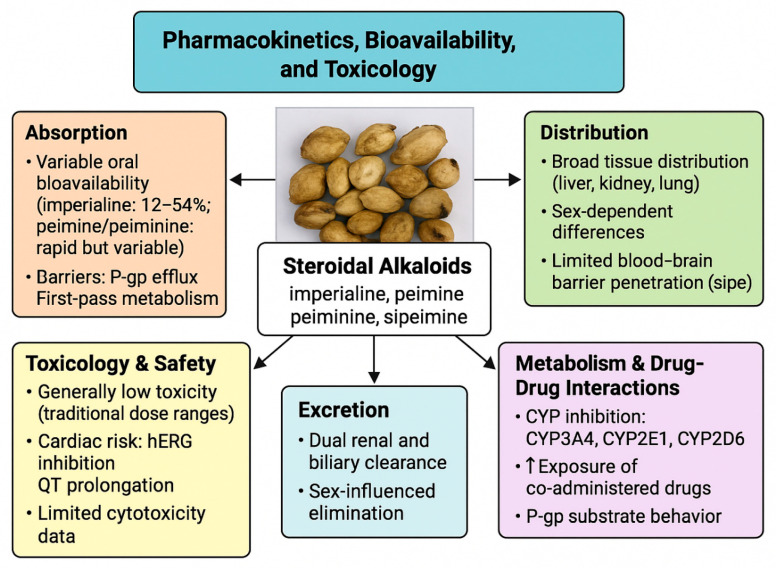
Pharmacokinetics, bioavailability, and toxicological characteristics of *F. pallidiflora* steroidal alkaloids. Absorption is constrained by variable oral bioavailability and barriers such as P-glycoprotein efflux and first-pass metabolism. Distribution occurs broadly across tissues, with sex-dependent variation and limited brain penetration. Metabolism involves CYP3A4, CYP2E1, and CYP2D6 inhibition, with potential drug–drug interactions and altered exposure of co-administered compounds. Excretion follows dual renal and biliary routes with sex-influenced clearance. Toxicological signals include generally low toxicity at traditional doses but a risk of hERG channel inhibition and QT prolongation, underscoring the need for safety evaluation in preclinical and clinical studies.

Despite long-standing traditional use, systematic pharmacokinetic data for *F. pallidiflora* compounds are limited. Existing studies suggest variable and often modest oral bioavailability for key steroidal alkaloids, with transporter- and enzyme-mediated barriers that complicate translation. Furthermore, species-specific toxicity data remain sparse, underscoring the need for in vivo safety evaluations before clinical adoption (Figure 7).

### 7.1. Absorption and Oral Bioavailability

Most ADME evidence comes from the major isosteroidal alkaloids shared across *Fritillaria* spp. notably imperialine (verticine), peimine, peiminine, and sipeimine all present in *F. pallidiflora* [74]. Across rodent studies, imperialine shows dose-dependent, method-sensitive oral bioavailability: an early HPLC-ELSD study at a high oral dose (~100 mg·kg^−1^) reported ~12% F, whereas a later LC–MS/MS study at lower doses (1–10 mg·kg^−1^) estimated ~31–54% F in rats, highlighting formulation/dose effects and analytical sensitivity [75]. For peimine/peiminine, validated LC–MS/MS assays in rats and beagle dogs enabled PK profiling after oral dosing, generally supporting rapid absorption with multi-compartment behavior but no consensus on high absolute F, and interspecies variation is evident [76]. From a physicochemical standpoint, cevanine-type steroidal alkaloids are polyhydroxylated, weakly basic molecules with relatively high polar surface area and moderate lipophilicity. This profile generally predicts limited passive membrane permeability, a propensity for interaction with efflux transporters such as P-glycoprotein, and substantial first-pass metabolism—features that are consistent with the modest and variable oral bioavailability reported for imperialine, peimine, peiminine, and sipeimine. Consequently, formulation strategies and dose levels can markedly influence systemic exposure.

Mechanistically, intestinal transport limits are implicated. Caco-2 and in vitro intestinal models indicate peimine is a P-glycoprotein (P-gp) substrate with active, efflux-influenced transport, offering a plausible explanation for constrained exposure and variability [77]. Related work on imperialine also points to permeability constraints in gut models. Together, these data support the view that efflux transport and first-pass metabolism shape the oral disposition of Beimu alkaloids.

### 7.2. Distribution

Tissue distribution has been characterized for select alkaloids. In rats, sex-dependent PK and broad tissue distribution were reported for peimine and peiminine, with quantification in plasma, tissues, urine, and feces, underscoring potential renal and biliary routes of elimination [78]. For sipeimine, a recent rat study found rapid absorption, slow elimination, ~40% oral bioavailability, ~30% plasma protein binding, and distribution to most organs with minimal brain exposure consistent with the high polarity of these alkaloids and efflux at the blood–brain barrier [79], support the view that efflux transport and first-pass metabolism shape the oral disposition of Beimu alkaloids (Figure 7).

### 7.3. Metabolism and Drug–Drug Interaction (DDI) Liabilities

Enzyme studies point to CYP involvement in alkaloid clearance. Peimine inhibits CYP3A4 in rat liver microsomes (time-dependent component reported), and also inhibits CYP2E1 and CYP2D6 in vitro, indicating DDI potential with co-medications that are CYP substrates [80]. In a rat DDI study, pretreatment with peimine significantly increased paeoniflorin exposure (↑C_max, ↑AUC, prolonged t_1_/_2_), consistent with CYP3A4 inhibition and altered intestinal transport; these findings are relevant for multi-herb formulae containing Beimu [81]. On the transporter side, P-gp substrate behavior (Section 5.1) provides a second axis for absorption-phase DDIs (Figure 7).

### 7.4. Excretion

Although comprehensive mass-balance studies are lacking for *F. pallidiflora* itself, urine and feces collection in the peimine/peiminine rat work implies dual renal and biliary excretion, with profiles influenced by sex [82]. Targeted metabolite identification has begun (e.g., for sipeimine in rat plasma and urine), but metabolic pathways for the broader *F. pallidiflora* alkaloid pool remain to be mapped systematically (Figure 7).

### 7.5. Toxicology and Safety Considerations

Genus-level safety summaries portray Bulbus *Fritillariae* as low-toxicity within traditional dose ranges, though rigorous species-specific toxicology for *F. pallidiflora* is limited [83]. A key cardiac safety signal is hERG channel inhibition by peimine, with IC_50_ ~40–44 μM in patch-clamp assays mechanistically linked to QT prolongation risk at sufficiently high exposures [84]. While historical use suggests a favorable safety margin, these in vitro cardiotoxicity flags justify exposure-aware dosing and in vivo ECG monitoring in preclinical development, especially for concentrated extracts or purified monomers. Limited in vitro cytotoxicity testing in macrophage models (RAW 264.7) found no cell viability loss up to 25 μg·mL^−1^ for several major alkaloids, yet this does not substitute for repeat-dose tox, safety pharmacology, and reproductive tox programs (Figure 7).

With respect to oral safety, a comparative acute toxicity study of ethanol extracts from *F. cirrhosa* and *F. pallidiflora* bulbs in mice reported an oral LD_50_ of approximately 213.6 g·kg^−1^ body weight for Bulbus *Fritillariae pallidiflorae*, indicating very low acute toxicity at clinically relevant doses and a wide margin between traditional dose ranges and lethal exposure levels. However, similar to other Beimu drugs, very high doses or misuse can still elicit neurological and respiratory symptoms, and chronic safety data for *F. pallidiflora* preparations remain limited. Taken together, these findings support the traditional view that Yi Beimu is ‘slightly cold and of low toxicity’, yet they also highlight the need to define species-specific no-observed-adverse-effect levels (NOAELs) and to establish safe exposure ranges for modern concentrated extracts and purified alkaloids.

In traditional Chinese medicine, Bulbus *Fritillariae* is regarded as slightly cold in nature and of relatively low toxicity; nevertheless, classical texts and modern toxicological studies of related species (e.g., *F. thunbergii* and *F. cirrhosa*) document that excessive doses or misidentification can lead to neurological symptoms, respiratory distress, and in severe cases convulsions and respiratory failure. Yi Beimu therefore exemplifies the ‘fine line between medicine and poison’: within recommended dose ranges it is safe and therapeutically beneficial, but concentrated extracts, long-term high-dose use, or combination with other CYP-modulating drugs could increase the risk of cardiotoxicity (via hERG inhibition), central nervous system symptoms, or herb–drug interactions. Clarifying species-specific NOAELs, target-organ toxicity, and chronic exposure effects in well-designed in vivo studies is an urgent prerequisite for modern dose optimization and rational clinical use.

### 7.6. Practical Implications and Gaps

Exposure hurdles: Efflux (P-gp) and CYP metabolism constrain oral exposure; bioavailability appears dose-, formulation-, and species-dependent [85].

Distribution limits: Minimal brain penetration reported for sipeimine aligns with BBB efflux and alkaloid polarity; targeting lung/liver tissues may be more tractable [86].

DDI risk management: CYP3A4/CYP2D6/CYP2E1 inhibition and P-gp interactions argue for careful co-medication assessment in formulae containing Yi Beimu [87].

Safety: hERG liability for peimine should be prioritized in in vivo QTc studies; species-specific NOAELs, target-organ tox, and chronic exposure data for *F. pallidiflora* preparations are needed [88].

The available ADME/Tox literature largely at the alkaloid level supports the pharmacological plausibility of Yi Beimu while revealing exposure limits, DDI liabilities, and cardiac safety flags that must be addressed. Targeted work in *F. pallidiflora* should couple bioavailability-enhancing formulations with PK-guided efficacy and comprehensive safety pharmacology to enable confident translation.

## 8. Conservation, Cultivation, and Sustainable Use

**Figure 8:**
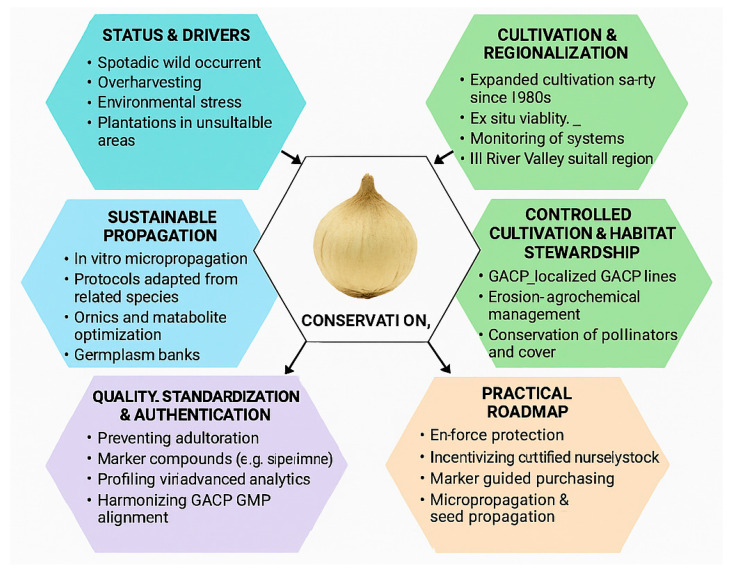
Conservation, cultivation, and sustainable use of *F. pallidiflora*. The figure integrates six thematic components: (i) status and resource pressure, including overharvesting, environmental stress, and unsuitable plantations; (ii) cultivation and regionalization, with the Ili River Valley as the core production zone and expanded ex situ cultivation; (iii) sustainable propagation strategies such as in vitro micropropagation, germplasm banking, and omics-assisted optimization; (iv) controlled cultivation and habitat stewardship through GACP adoption, soil–ecological management, and pollinator conservation; (v) quality standardization and authentication using pharmacopoeial markers, chemometric profiling, and GMP alignment; and (vi) a practical roadmap that combines habitat protection, nursery-based seedling supply, marker-guided purchasing, and propagation scaling. Together, these strategies outline an integrated framework for balancing ecological conservation with medicinal resource security.

The increasing demand for *F. pallidiflora* has placed immense pressure on wild populations, leading to its designation as a nationally protected species in China. While cultivation programs in Xinjiang and Inner Mongolia have emerged as important conservation strategies, ecological sustainability and quality control remain pressing challenges. Ensuring both the protection of natural habitats and the development of reliable cultivation systems is essential to safeguard Yi Beimu for future medicinal use (Figure 8).

### 8.1. Status and Drivers of Resource Pressure

Wild populations of *F. pallidiflora* in Xinjiang now occur only sporadically in middle-mountain shrub habitats, with near disappearance from the low-mountain belts where the species was once more abundant [89]. This decline reflects the dual pressures of overharvesting and environmental stress, both of which have intensified as market demand has increased. The expansion of the domestic herbal medicine market has also encouraged indiscriminate cultivation, with some farmers establishing plantations in ecologically unsuitable areas. Such practices risk soil–microclimate mismatch and ultimately compromise the quality and consistency of bulb resources [90]. Although Beimu drugs as a whole face well-documented issues of supply shortage and substitution, species-specific reporting on *F. pallidiflora* is still scattered [91]. Nevertheless, its restricted native distribution in northwestern Xinjiang particularly in the Ili, Altay, and Tacheng regions together with its official protected status underscores the urgency of establishing managed and sustainable sourcing strategies [92]. This restricted distribution, combined with official protected status, underscores the urgency of establishing managed and sustainable sourcing strategies (Figure 8).

### 8.2. Progress in Cultivation and Regionalization

Efforts to domesticate *F. pallidiflora* began in the 1960s, and the species is now cultivated across a wider geographic range, including Inner Mongolia, Gansu, Shaanxi, Henan, and Shandong provinces [93]. Among these, the Ili River Valley in Xinjiang remains the primary production area, largely due to its cool, humid, and high-altitude climate that is particularly favorable for bulb formation and alkaloid accumulation [94]. Reports in the *Flora of China* and related agronomic studies confirm that ex situ cultivation is feasible, provided that careful attention is paid to soil conditions, temperature regimes, and photoperiod [95]. However, production systems require more precise agronomic zoning and ongoing monitoring to prevent yield–quality trade-offs. Without such measures, expansion may generate larger harvests but at the cost of the pharmacological efficacy of the bulbs. Without careful agronomic zoning, expansion may increase yields but compromise bulb quality and pharmacological efficacy (Figure 8).

### 8.3. Sustainable Propagation: Tissue Culture and Seed Systems

To relieve harvesting pressure on wild populations and ensure uniformity in cultivated material, modern propagation techniques are essential [96]. In vitro micropropagation provides an efficient method to generate pathogen-free, genetically stable bulblets year-round. Although species-specific protocols for *F. pallidiflora* remain limited, propagation methods developed for related *Fritillaria* species, such as *F. cirrhosa*, *F. imperialis*, and *F. meleagris*, offer transferable frameworks [97]. Techniques involving bulb-scale or leaf explants, the use of cytokinins such as BAP and KIN in combination with low doses of auxins, and cold pretreatments to break dormancy have been successfully applied in these species and can be adapted for Yi Beimu [98]. Modern micropropagation and seed-based approaches provide scalable solutions for reducing harvesting pressure while maintaining chemotypic integrity (Figure 8). Recent reviews also emphasize the role of omics-assisted propagation, which can optimize not only plantlet yield but also metabolite production, ensuring that imperialine- and peimine-rich chemotypes are maintained [99]. For long-term sustainability, complementary strategies include establishing regional nurseries to scale micropropagated bulblets for farmers, developing germplasm banks that capture eco-geographic diversity, and combining propagation with habitat restoration or rotational seed collection to preserve genetic integrity [100].

### 8.4. Controlled Cultivation and Habitat Stewardship

While expanding cultivation capacity is necessary, it must be coupled with ecological safeguards. The adoption of Good Agricultural and Collection Practices (GACP) tailored for medicinal geophytes is critical to maintaining both resource quality and ecological balance [101]. GACP guidelines should cover site selection, soil preparation, irrigation practices, and responsible use of agrochemicals, harvest timing, and post-harvest handling [102]. International frameworks such as the WHO-GACP and the updated EMA-HMPC GACP (2025 revision) provide useful standards that can be localized to Yi Beimu production zones [103]. In addition to agronomic measures, ecological stewardship such as erosion control, maintenance of shrub–grass cover, and conservation of pollinator habitats will be vital given the mountain-steppe ecosystems where *F. pallidiflora* thrives [104]. Localized GACP protocols and ecological stewardship, including erosion control and pollinator habitat maintenance, are critical for balancing production with ecosystem integrity (Figure 8).

### 8.5. Quality Standardization and Authentication

As cultivation expands, ensuring quality consistency is a major challenge. Quality drift and adulteration, often due to confusion between *F. pallidiflora* and *F. cirrhosa* bulbs, necessitate a multi-tiered standardization system [105]. At the pharmacopoeial level, the Chinese Pharmacopoeia already lists *F. pallidiflora* and specifies marker compounds such as sipeimine-3β-D-glucoside and sipeimine. Advanced UPLC/LC–MS methods have further improved resolution by targeting additional markers like peimisine and yibeinoside A. Chemometric authentication studies have also demonstrated the ability to distinguish *F. cirrhosa* from *F. pallidiflora* using specific isosteroidal alkaloid profiles, which is critical for preventing substitution and fraud in the herbal market [106]. Finally, harmonizing farm-level GACP with GMP in processing facilities and aligning with buyer specifications, such as AHPA’s GACP-GMP standards, will ensure traceability, safety, and consistent alkaloid profiles across production batches. Advanced chemometric authentication and marker-guided quality systems are essential to prevent substitution and maintain consistent pharmacological profiles (Figure 8).

### 8.6. Practical Roadmap

In practice, the conservation and sustainable use of *F. pallidiflora* will require a dual approach. First, ecological protection must be strengthened by enforcing its protected status, designating no-take conservation zones, and supporting community co-management of shrub–meadow habitats [107]. Second, cultivation must replace wild digging through the widespread adoption of certified nursery stock, incentivized by price premiums for pharmacopoeia-compliant material [108]. Third, quality must be standardized through marker-guided purchasing, lot-level chemotyping, and chemical authentication to discourage substitution [109]. Finally, propagation programs should be scaled up, using both micropropagation and seed systems, to ensure genetic diversity and the maintenance of desired chemotypes [110]. An integrated roadmap combining protection enforcement, certified nursery stock, and market-guided procurement ensures sustainable use while relieving pressure on wild populations (Figure 8).

In summary, the sustainable future of Yi Beimu depends on balancing conservation with cultivation. Protecting wild habitats, guiding cultivation to climatically suitable regions such as the Ili Valley, and embedding robust quality assurance throughout the supply chain will be critical. Only through this integrated strategy can pressure on wild populations be alleviated while ensuring that *F. pallidiflora* continues to provide consistent therapeutic benefits as both a traditional and modern medicinal resource.

## 9. Challenges and Future Perspectives

Future research must move beyond compound cataloging towards mechanistic elucidation and translational validation. Integrating ethnopharmacological wisdom with modern drug discovery approaches such as metabolomics, systems pharmacology, and clinical testing will be essential to unlock the full therapeutic value of *F. pallidiflora*.

### 9.1. Knowledge Gaps

Despite a robust record of traditional use and steadily expanding phytochemical reports, mechanistic studies on *F. pallidiflora* remain limited. Most work has focused on structural elucidation of steroidal alkaloids, saponins, and polysaccharides, with pharmacological testing confined largely to in vitro systems (e.g., macrophage NO inhibition, radical scavenging, tracheal relaxation assays). Few in vivo models have validated efficacy in complex disease contexts, and no controlled clinical trials have been conducted to confirm safety, pharmacokinetics, or therapeutic benefit in humans. This evidence gap not only restricts translation into modern practice but also leaves the long-term safety profile of concentrated extracts and isolated compounds unresolved.

### 9.2. Methodological Needs

Advancing the field requires shifting from descriptive to mechanistic research frameworks. First, bioactivity-guided isolation should be prioritized to link specific chemical classes (e.g., imperialine derivatives, pallidiflosides) with defined pharmacological outcomes. Second, omics technologies including metabolomics, transcriptomics, and proteomics—can map the pathways influenced by *F. pallidiflora* extracts, revealing multi-target interactions and network effects consistent with traditional decoction use. Third, systems pharmacology and computational docking approaches could help predict synergistic or antagonistic relationships among alkaloids, saponins, and polysaccharides. Finally, integrated pharmacokinetic–pharmacodynamic (PK–PD) studies are urgently needed to understand absorption barriers, metabolism, transporter interactions, and dose–response relationships, laying the foundation for rational formulation and clinical trial design.

### 9.3. Drug Discovery Potential

The chemical diversity of *F. pallidiflora* offers promising leads for drug discovery. Cevanine-type alkaloids such as imperialine and peimine have demonstrated airway-relaxant and anti-inflammatory activities, suggesting potential for respiratory drug development. Meanwhile, novel saponins (pallidiflosides A–C, D–I) and unique glycoalkaloids (yibeinones) expand the structural landscape, some already showing cytotoxic or immunomodulatory properties in vitro. If optimized for bioavailability and safety, these molecules could serve as scaffolds for next-generation antitussive, anti-inflammatory, or anticancer agents. Equally important is the prospect of synergistic formulations, where multiple compounds mimic the complexity of traditional decoctions but with modern quality control.

### 9.4. Toward Sustainable and Translational Development

Sustainability challenges intersect with scientific gaps. Rising demand for Yi Beimu risks overharvesting and threatens wild populations; hence linking conservation with research pipelines is crucial. Future drug discovery and pharmacological validation efforts must be grounded in sustainably cultivated or tissue-culture-derived material to ensure both ecological and commercial viability. Bridging traditional knowledge systems with rigorous biomedical validation will also enhance cultural legitimacy and clinical acceptability.

In conclusion, the future of *F. pallidiflora* research lies in integrating traditional use with modern science, progressing from compound inventories to mechanism-driven, clinically validated interventions, and embedding these efforts within a sustainable cultivation framework. This integrated trajectory has the potential not only to safeguard a culturally significant herb but also to deliver novel therapeutics for pressing global health challenges.

## 10. Conclusions

This review highlights *F. pallidiflora* as an ethnomedicinally valuable but underexplored species within the broader Beimu group of traditional Chinese medicines. Its long-standing role in treating cough, asthma, and phlegm-related disorders is supported by the presence of diverse steroidal alkaloids, saponins, and polysaccharides that display antitussive, anti-inflammatory, antioxidant, and other pharmacological activities. Recent studies have expanded the phytochemical inventory of *F. pallidiflora* to include unique cevanine-type alkaloids and novel saponins, underscoring its potential as a source of bioactive compounds with therapeutic promise.

Despite these advances, current evidence remains fragmented and largely confined to in vitro assays, with limited mechanistic exploration and almost no translational validation. Systematic studies on pharmacokinetics, metabolism, and toxicity are still lacking, and no clinical trials have yet confirmed safety or efficacy in humans. These gaps must be addressed through integrated research approaches that combine bioactivity-guided isolation, omics-based pathway analysis, and in vivo pharmacological models.

At the same time, the species faces ecological pressures from overharvesting and habitat loss, necessitating urgent attention to conservation and sustainable cultivation. Controlled propagation, germplasm preservation, and standardized quality assurance will be critical to ensure both ecological stewardship and reliable medicinal supply.

In summary, while the phytochemistry and pharmacological potential of *F. pallidiflora* are promising, its development as a modern therapeutic resource depends on filling critical gaps in mechanistic, pharmacokinetic, and clinical research, alongside sustainable resource management. If these challenges are met, Yi Beimu may transition from a traditional remedy into a scientifically validated and globally relevant medicinal plant.

## Figures and Tables

**Table 1:** Representative constituents of *F. pallidiflora* (selected examples).

Compound (Example)	Class	Noted Activity/Use	Reference
Imperialine (verticine)	Steroidal isosteroidal alkaloid	Antitussive/bronchodilatory; hallmark Beimu alkaloid	[8]
Peimine/Peiminine	Steroidal isosteroidal alkaloids	Respiratory and anti-inflammatory signals in genus; QC markers	[49]
Peimisine	Steroidal alkaloid	QC marker; co-occurs with sipeimine	[50]
Sipeimine	Steroidal alkaloid	QC/PK marker; UPLC–MS/MS quantification and metabolism mapping	[51]
Yibeinone F (24-OH cevanine)	New steroidal alkaloid	Expands *F. pallidiflora* alkaloid chemotype space	[52]
Ring-B-seco isosteroidal alkaloid (unnamed) + 5 SGAs	Rare glycoalkaloids	Structural novelty; potential bioactivities	[53]
Pallidiflosides A–C	Steroidal saponins	Chemotaxonomic markers; reported cytotoxicity (in vitro)	[54]
Additional saponins (5 new + 7 known)	Steroidal saponins	Cytotoxic against C6/HeLa (compound-dependent)	[55]
FPSP-H2-1 (acidic fraction)	Polysaccharide	Potent radical scavenging (ABTS/DPPH)	[56]
Imperialine-β-D-glucoside	Alkaloid glycoside	Historical isolation from bulbs; chemotaxonomic interest	[57]

## Data Availability

Not applicable.
